# Targeting STAT3 to Modulate the Effectiveness of Cardioprotective Conditioning Strategies Against Myocardial Ischemia–Reperfusion Injury in Subjects With Diabetic Cardiomyopathy

**DOI:** 10.1111/jcmm.71418

**Published:** 2026-10-23

**Authors:** Chunyan Wang, Wangli Tian, Saman Saedi, Hengzhen Cui, Danyong Liu, Qinjun Chu, Dongwei Yang, Zhengyuan Xia

**Affiliations:** ^1^ Institute of Trauma and Metabolism Zhengzhou Central Hospital Affiliated With Zhengzhou University Zhengzhou China; ^2^ Department of Anesthesiology Shenzhen Maternity and Child Healthcare Hospital Shenzhen Guangdong China; ^3^ Department of Anesthesiology and Perioperative Medicine Zhengzhou Central Hospital Affiliated With Zhengzhou University Zhengzhou China; ^4^ Cell Research and Transformation Center Zhengzhou Central Hospital Affiliated With Zhengzhou University Zhengzhou China; ^5^ Department of Cardiology Zhengzhou Central Hospital Affiliated With Zhengzhou University Zhengzhou China; ^6^ State Key Laboratory of Pharmaceutical Biotechnology, Department of Medicine The University of Hong Kong Hong Kong China

**Keywords:** cardioprotection, diabetes mellitus, diabetic cardiomyopathy, ischemic conditioning, myocardial ischemia–reperfusion injury, STAT3

## Abstract

Myocardial ischemia–reperfusion injury (MIRI) is a major cause of cardiovascular morbidity and mortality. Reperfusion, although essential for myocardial restoration, can paradoxically exacerbate injury through oxidative stress, calcium overload, mitochondrial permeability transition pore opening and inflammatory infiltration. The vulnerability to MIRI is substantially amplified in subjects with diabetes mellitus, where chronic hyperglycemia, insulin resistance, and the resulting oxidative stress and low‐grade inflammation impair endogenous cardioprotective mechanisms and worsen postinfarction outcomes. Ischemic conditioning strategies, including preconditioning and postconditioning, serve as powerful innate methods to mitigate MIRI by exposing the myocardium to brief, sublethal ischemic episodes that activate crucial prosurvival signalling pathways. Among these pathways, the survivor activating factor enhancement pathway, which is mediated predominantly by signal transducer and activator of transcription 3 (STAT3), and the reperfusion injury salvage kinase pathway, which involves AKT and ERK, are particularly important. However, in many models of diabetes, diabetes‐induced STAT3 dysfunction may disrupt these protective pathways, often making conditioning stimuli less effective. The precise mechanisms underlying this impairment remain incompletely understood. Additionally, paradoxical changes in STAT3 expression have been reported, and key posttranslational modifications of STAT3 have not yet been investigated in the context of diabetic MIRI. This review provides a critical appraisal of the role, regulatory mechanisms and therapeutic potential of STAT3 in ischemic conditioning in patients with diabetes and discusses strategies aimed at restoring cardioprotection in this high‐risk population.

**Abbreviations**
AAV: Adeno‐associated virus
AGE: Advanced glycation end product
AKT: Protein kinase B
CABG: Coronary artery bypass grafting
CT‐1: Cardiotrophin‐1
DPP‐4: Dipeptidyl peptidase‐4
ERK: Extracellular signal‐regulated kinase
ETC: Electron transport chain
GAS: Gamma‐activated sequence
GLP‐1 RA: Glucagon‐like peptide‐1 receptor agonist
GSK‐3β: Glycogen synthase kinase‐3 beta
IL‐6: Interleukin‐6
IPC: Ischemic preconditioning
JAK: Janus kinase
LIF: Leukaemia inhibitory factor
m6A: N6‐methyladenosine
MIRI: Myocardial ischemia–reperfusion injury
mitoSTAT3: Mitochondrial STAT3
MnSOD: Manganese superoxide dismutase
mPTP: Mitochondrial permeability transition pore
NOX: NADPH oxidase
P: Posttranslational modification
PCI: Percutaneous coronary intervention
PGC‐1α: Peroxisome proliferator‐activated receptor gamma coactivator 1‐alpha
PI3K: Phosphoinositide 3‐kinase
PIAS: Protein inhibitor of activated STATs
PIP3: Phosphatidylinositol‐3,4,5‐trisphosphate
RIPC: Remote ischemic preconditioning
RISK: Reperfusion injury salvage kinase
ROS: Reactive oxygen species
SAFE: Survivor activating factor enhancement
SGLT2: Sodium–glucose cotransporter 2
SIRT1: Sirtuin 1
SMYD2: SET and MYND domain‐containing protein 2
SOCS: Suppressor of cytokine signalling
STAT3: Signal transducer and activator of transcription 3
STAT5: Signal transducer and activator of transcription 5
STEMI: ST‐segment elevation myocardial infarction
SUMO: Small ubiquitin‐like modifier
TNF‐α: Tumour necrosis factor‐alpha
ΔΨm: Mitochondrial membrane potential

## 1 Introduction

Myocardial ischemia–reperfusion injury (MIRI) is a major clinical challenge and a leading contributor to cardiovascular morbidity and mortality worldwide [1]. As comprehensively reviewed, the pathophysiology of MIRI encompasses both coronary vascular and myocardial components; however, despite decades of research, the translation of mechanistic insights into clinical practice remains a major challenge [2]. At the tissue level, coronary occlusion induces energy depletion, ionic imbalance and hypoxia in cardiomyocytes. Although reperfusion is essential for myocardial salvage, it paradoxically triggers additional injury that may exceed the initial degree of ischemic damage [3].

Despite the unequivocal benefit of timely reperfusion, its translation into improved clinical outcomes is hampered by the fact that patients who present with acute myocardial infarction are often elderly and have multiple comorbidities and concomitant medications—factors that profoundly modify the pathophysiology of MIRI and the efficacy of cardioprotective interventions [4]. Among these, diabetes mellitus stands out as a particularly potent modifier: it not only exacerbates myocardial injury but also renders the heart refractory to endogenous protective signals, a phenomenon closely linked to dysfunction of key survival pathways [5].

The clinical significance of this vulnerability is magnified by the global burden of diabetes, which affects more than 500 million adults worldwide [6]. Compared with their non‐diabetic counterparts, diabetic patients with acute myocardial infarction have larger infarct sizes, worse ventricular function and higher mortality rates, with particularly adverse outcomes in women [7, 8]. These poor outcomes are largely attributable to metabolic and molecular alterations associated with diabetic cardiomyopathy, which profoundly impair endogenous cardioprotective mechanisms.

Among the endogenous strategies capable of mitigating MIRI, ischemic conditioning, including pre, peri and postconditioning, has emerged as a powerful non‐pharmacological approach. By subjecting the myocardium to brief, nonlethal ischemic episodes, conditioning induces a state of heightened tolerance to subsequent prolonged ischemia, operating through both an early phase (minutes) and a delayed phase (24–72 h), and significantly reduces infarct size and improves contractile recovery in non‐diabetic subjects [9]. These protective interventions activate multiple pro‐survival signalling cascades, most notably the survivor activating factor enhancement (SAFE) pathway—initiated by cytokines such as TNF‐α and IL‐6 via JAK kinases, leading to STAT3 phosphorylation and activation and the reperfusion injury salvage kinase (RISK) pathway, which primarily involves AKT and ERK to safeguard mitochondrial integrity and exert anti‐apoptotic and anti‐inflammatory effects.

In the diabetic heart, however, conditioning‐mediated protection is markedly attenuated [10, 11, 12]. A central mechanism underlying this complication is the impairment of the SAFE pathway—specifically, diabetes‐induced dysfunction of STAT3, which in certain experimental settings disrupts the intrinsic protective cascade and may render conditioning stimuli ineffective, thereby potentially increasing vulnerability to reperfusion damage. A focused understanding of STAT3 regulation in the diabetic myocardium is critically needed to translate mechanistic insights into targeted therapies for this high‐risk population. Accordingly, this review aims to elucidate the precise role, regulatory mechanisms and therapeutic potential of STAT3 in ischemic conditioning in diabetic hearts.

Given the narrative scope of this review, we nevertheless employed a structured literature search to ensure comprehensive coverage and transparency. We performed a structured search of PubMed/MEDLINE, Web of Science and Scopus for English‐language articles, reviews and meta‐analyses, using combinations of keywords including ‘remote ischemic conditioning,’ ‘STAT3,’ ‘cardioprotection,’ ‘myocardial ischemia/reperfusion,’ ‘diabetic cardiomyopathy,’ ‘mitochondria,’ and ‘extracellular vesicles.’ Two authors independently screened titles and abstracts, with disagreements resolved by a third author, and reference lists of included articles were manually cross‐checked. This search strategy was not intended to perform a quantitative meta‐analysis but rather to comprehensively inform the narrative synthesis presented herein.

## 2 Enhanced Susceptibility to MIRI in Diabetes Patients: Pathophysiology and Clinical Evidence

MIRI is driven by complex pathophysiological mechanisms that remain incompletely understood [13, 14]. This complexity is underscored by the interplay of multiple interacting pathways—ranging from oxidative stress and calcium overload to mitochondrial dysfunction and inflammatory infiltration, whose relative contributions may vary depending on the underlying metabolic state [2]. The initial ischemic insult depletes cellular energy and disrupts ionic homeostasis, thereby creating conditions that amplify subsequent damage. Reperfusion, while indispensable for myocardial salvage, paradoxically triggers a cascade of injurious events, such as a burst of reactive oxygen species (ROS) [15], calcium overload [16], a profound inflammatory response characterized by neutrophil infiltration and proinflammatory cytokine release [3] and irreversible opening of the mitochondrial permeability transition pore (mPTP). mPTP opening decreases the mitochondrial membrane potential (ΔΨm) and the proton gradient, disrupting respiratory function, impairing ATP synthesis and ultimately leading to cardiomyocyte death [17, 18, 19, 20].

Diabetes profoundly amplifies this injury cascade. The cardinal features of diabetes—hyperglycemia and insulin resistance—drive chronic low‐grade inflammation, oxidative stress and mitochondrial dysfunction [21, 22], creating a ‘hostile baseline environment’ that depletes endogenous protective reserves [23]. Notably, diabetes significantly impairs pro‐survival signalling cascades, including the RISK and SAFE pathways and exacerbates mPTP opening [24]. These metabolic and molecular alterations in diabetic cardiomyopathy fundamentally disrupt the intracellular signalling that mediates endogenous cardioprotection, with STAT3 dysfunction emerging as a central mechanism. As illustrated in Figure 1, metabolic and inflammatory stress in the diabetic heart impairs STAT3‐dependent SAFE signalling, attenuates endogenous protection and heightens cardiac susceptibility to MIRI.

**FIGURE 1:**
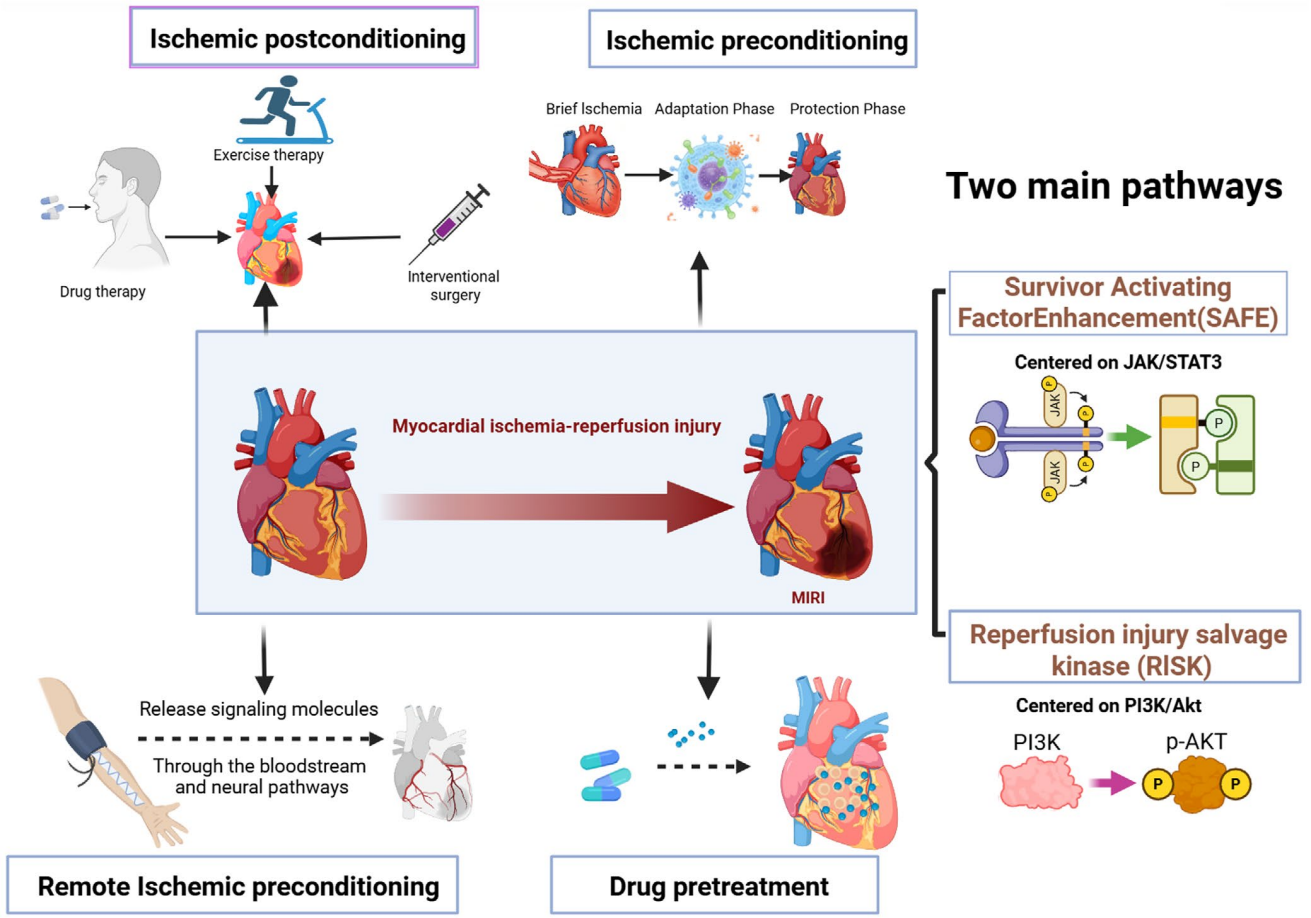
The conditioning effect on MIRI. This includes postischemic treatment, ischemic preconditioning, remote ischemic preconditioning and drug pretreatment. Conditioning primarily confers a cardioprotective effect through the SAFE and RISK pathways. This figure was created at biorender.com.

Clinical evidence supports a worse prognosis for diabetic patients following myocardial infarction. In a nationwide analysis of more than 2.5 million acute myocardial infarction (AMI) patients, those with either type 1 (T1D) or type 2 diabetes (T2D) experienced significantly higher in‐hospital mortality and major adverse cardiovascular and cerebrovascular events (MACCEs) than non‐diabetic individuals did [25]. Similarly, in the DIAMOND registry, compared with non‐diabetic patients, diabetic patients had significantly higher 2‐year rates of MACEs (8.0% vs. 3.7%), all‐cause death (3.9% vs. 1.4%) and recurrent MI (2.8% vs. 1.2%) after AMI [23]. Cardiovascular magnetic resonance studies have demonstrated that compared with normoglycemic patients, diabetic patients sustain significantly larger myocardial infarcts [26]. In the context of ST‐elevation myocardial infarction (STEMI), diabetic patients have a mortality rate twice that of non‐diabetic patients [27], and they face higher risks of in‐hospital mortality and cerebrovascular events when AMI is complicated by cardiogenic shock [28]. Furthermore, after AMI, diabetic patients with heart failure have a substantially worse long‐term prognosis than non‐diabetic patients with heart failure do [29]. Collectively, these clinical observations underscore that diabetes not only increases acute ischemic vulnerability but also leads to persistently worse outcomes following myocardial infarction.

Beyond the abovementioned direct prognostic disadvantages, compelling clinical and translational evidence indicates that diabetes specifically disrupts the endogenous cardioprotective machinery, rendering the diabetic heart refractory to ischemic conditioning stimuli. A landmark clinical study by Ishihara et al. enrolled 611 patients with a first acute anterior wall AMI and revealed that prodromal angina occurring within 24 h before infarction (acting as an endogenous IPC stimulus) significantly limited infarct size, enhanced recovery of the left ventricular ejection fraction and reduced in‐hospital mortality in nondiabetic patients. In sharp contrast, diabetic patients derived no benefit from prodromal angina for any of these endpoints, strongly suggesting that diabetes prevents ischemic preconditioning [30]. This conclusion is further supported by ex vivo studies using human myocardium: trabeculae obtained from patients with diabetes failed to respond to preconditioning stimuli, whereas those from non‐diabetic patients were robustly protected [31, 32].

The efficacy of remote ischemic preconditioning (RIPC) is also compromised in patients with diabetes. In patients undergoing coronary artery bypass grafting, sulfonylureas abolished the cardioprotective effects of RIPC [33], and the humoral factor released by RIPC failed to protect the heart in diabetic patients with upper‐limb neuropathy, indicating that intact neural pathways are required for RIPC‐induced cardioprotection [34]. The large‐scale CONDI‐2/ERIC‐PPCI trial, which evaluated RIPC in STEMI patients undergoing percutaneous coronary intervention, did not show improved clinical outcomes; notably, only approximately 12% of the enrolled patients had diabetes, and comorbidities and concomitant medications may have confounded the protective signal [35]. Together, these findings establish that diabetes mellitus not only exacerbates MIRI but also, more importantly, renders the heart refractory to both endogenous and remote conditioning strategies. This cardioprotection resistance aligns closely with dysfunction of the STAT3‐dependent SAFE pathway, as detailed in the following sections.

## 3 Molecular Mechanisms Underlying Increased Sensitivity to MIRI and Impaired STAT3 Cardioprotection in Diabetic Cardiomyopathy

The heightened vulnerability of the diabetic heart to MIRI and resistance to cardioprotective conditioning are driven by a constellation of molecular aberrations converging on STAT3 dysfunction. Preclinical investigations have focused on deciphering these mechanisms, revealing multiple reinforcing layers of STAT3 impairment, spanning transcriptional regulation, post‐translational modification, membrane microenvironment disruption and mitochondrial quality control.

When interpreting STAT3 signalling in diabetic MIRI, however, a critical distinction must be made between T1D and T2D models. T1D is characterized by absolute insulin deficiency, whereas T2D involves insulin resistance with hyperinsulinemia. While both models exhibit excessive cardiac FOXO1 activation and suppressed STAT3 phosphorylation—as demonstrated in rodent studies—the divergent metabolic milieus may disrupt insulin‐PI3K/JAK2‐STAT3 crosstalk through distinct molecular nodes. Currently, direct head‐to‐head comparative studies are scarce, and findings from one model are often generalized to the other without rigorous justification. Future research should explicitly specify the diabetes type in experimental designs and, ideally, conduct head‐to‐head comparisons to identify shared mechanisms versus disease‐specific targets for STAT3 restoration.

### 3.1 Dysregulation of Upstream Negative Regulators of STAT3 Signalling

Diabetes induces a state of chronic low‐grade inflammation and metabolic stress that profoundly alters the balance between STAT3‐activating and STAT3‐suppressing signals. Several lines of evidence have identified key negative regulators that are upregulated or hyperactivated in the diabetic myocardium, often contributing to attenuated STAT3 activation under chronic hyperglycemic conditions. Excessive activation of forkhead box O1 (FOXO1) in diabetic hearts directly antagonizes STAT3 signalling. FOXO1 physically associates with STAT3 and its hyperactivity suppresses STAT3 activation, leading to impaired mitophagy and mitochondrial dynamics. Selective pharmacological inhibition of FOXO1 with AS1842856 restored STAT3 activation and alleviated cardiac dysfunction, highlighting the causative role of FOXO1‐driven STAT3 suppression in diabetic cardiomyopathy [12]. This finding is particularly important, as it positions FOXO1 as a critical upstream node linking diabetes‐induced metabolic dysregulation to STAT3 dysfunction.

PH domain leucine‐rich repeat protein phosphatase 1 (PHLPP1) has emerged as an important negative regulator of AKT and STAT3 signalling. PHLPP1 levels were elevated in the adipose and muscle tissues of obese patients and contribute to insulin resistance [36]. Recent preclinical studies have demonstrated that pharmacological or genetic suppression of PHLPP1 protects against MIRI in diabetic mice by restoring STAT3 activation, suggesting that targeting PHLPP1 may overcome the ‘STAT3‐deficient’ state in diabetic hearts [37].

The JAK–STAT pathway is tightly regulated by protein tyrosine phosphatases (PTPs), which dephosphorylate and inactivate STAT3. Among these, T‐cell protein tyrosine phosphatase (TCPTP) has been identified as a direct negative regulator of STAT3. TCPTP can directly dephosphorylate STAT3, and its dysregulation in diabetes may thus impair STAT3 activation [38]. Additionally, myeloid PTP1B has been implicated in the negative regulation of STAT3/IL‐10 mediated anti‐inflammatory signalling in obesity, providing a potential mechanistic link between diabetes‐related inflammation and impaired STAT3‐dependent cardioprotection [39].

Chronic hyperglycemia drives the accumulation of advanced glycation end products (AGEs), which engage the receptor for AGEs (RAGE) to activate multiple stress pathways. Notably, AGEs have been shown to have complex effects on the JAK2/STAT3 axis. While acute activation of JAK2/STAT3 by AGEs may occur in some contexts, chronic AGE/RAGE signalling in diabetes contributes to inflammatory and fibrotic responses that may ultimately desensitize or dysregulate STAT3‐mediated protective signalling. Moreover, AGEs can promote pathological STAT3 phosphorylation patterns associated with adverse vascular remodelling, further complicating the role of STAT3 in the diabetic heart [40].

### 3.2 Oxidative Stress, Membrane Disruption and Mitochondrial Dysfunction as Converging Mechanisms of STAT3 Impairment

Diabetes‐induced hyperglycemia drives excessive ROS production through multiple sources, including mitochondrial electron transport chain (ETC) leakage, NADPH oxidase activation and AGE/RAGE signalling [41]. This oxidative burden attacks the cardiomyocyte at two interconnected fronts. First, ROS directly disrupt the membrane microenvironment by downregulating caveolin‐3 (Cav‐3), an essential scaffold for organizing JAK2/STAT3 signalling complexes, thereby blunting STAT3 phosphorylation in response to conditioning stimuli [42]. Second, ROS inflict structural and functional damage to mitochondria—impairing ETC efficiency, dissipating the mitochondrial membrane potential (ΔΨm), reducing ATP synthesis and sensitizing the permeability transition pore (mPTP) to calcium‐induced opening [43]. Mitochondrial injury, in turn, amplifies ROS production through a feedforward ‘ROS‐induced ROS release’ mechanism [43], perpetuating the oxidative burden.

This ROS‐mitochondria axis is further reinforced by hyperglycemia‐induced FOXO1 hyperactivity. FOXO1 physically associates with and suppresses STAT3 activation, while simultaneously impairing mitophagy, promoting mitochondrial fission and reducing fusion, collectively compromising mitochondrial quality control [12]. Consequently, STAT3 phosphorylation at both Tyr705 and Ser727 is reduced, mitochondrial STAT3 (mitoSTAT3) function is compromised, and the myocardial defence against ischemic stress is critically weakened. The downstream consequences of this integrated cascade are detailed in subsequent sections (see Section 4 for functional consequences on conditioning efficacy).

### 3.3 Metabolic and Insulin Signalling Crosstalk With STAT3

Insulin resistance, a core feature of T2D, profoundly affects STAT3 signalling. The PI3K/AKT pathway and the JAK2/STAT3 pathway exhibit bidirectional crosstalk such that impairment of one may compromise the other. Studies have shown that in some contexts, diabetes‐induced STAT3 dysfunction contributes to the failure of insulin to restore postconditioning cardioprotection. These findings suggest that the inability of insulin to confer myocardial protection in diabetic hearts may be attributed, at least in part, to STAT3‐mediated inhibition of PI3K signalling [44]. These findings underscore the intertwined nature of metabolic and survival signalling in the diabetic myocardium.

### 3.4 STAT3 in Immune Regulation: Macrophage Polarization and Inflammation

Beyond its cardiomyocyte‐intrinsic protective effects, STAT3 critically orchestrates the post‐infarction inflammatory response through its cell‐type‐specific functions. In infiltrating macrophages, STAT3 activation drives the transition from pro‐inflammatory M1 to reparative M2 polarization, a process essential for limiting infarct expansion and facilitating cardiac repair. In diabetes patients, this STAT3‐dependent polarization switch is delayed, contributing to persistent inflammation and adverse ventricular remodelling. Importantly, the role of STAT3 in the immune compartment is functionally distinct from its cardiomyocyte functions, yet both are interconnected through paracrine cytokine networks (e.g., IL‐10 feedback). Given the dual nature of STAT3, where acute activation promotes resolution but chronic overactivation may drive fibrosis—therapeutic strategies targeting inflammation in the diabetic heart must carefully balance these opposing outcomes, and future studies should delineate the temporal and cell‐type‐specific contributions of STAT3 to post‐MIRI remodelling.

Collectively, the molecular defects outlined in this section—ranging from negative regulator overexpression and oxidative membrane damage to immune dysregulation—converge to establish a persistently STAT3‐deficient state. This state directly explains why the SAFE and RISK pathways, which are the subject of the next section, fail to be activated by conditioning stimuli in the diabetic heart.

## 4 Cardioprotective and Prosurvival Signalling in Ischemic Conditioning: PI3K/AKT and JAK/STAT3

Ischemic conditioning is a potent endogenous protective strategy that enhances myocardial resilience to MIRI. Ischemic pre‐, peri‐ and postconditioning, as well as pharmacological conditioning, have revealed potent endogenous signalling pathways that can mitigate MIRI [3, 45, 46]. Among these pathways, the activation of the STAT3, AKT and ERK pathways is critically involved [30] and occurs either synergistically or independently.

In our clinical study involving 112 children with tetralogy of Fallot who underwent radical open cardiac surgery using cardiopulmonary bypass (CPB), 55 patients were randomized to the remote ischemia preconditioning (RIPC) group, and 57 were randomized to the control group. We found that RIPC conferred cardioprotective effects, which were coincident with increased cardiac STAT3 activation [31]. The RIPC protocol involved three cycles of 5‐min lower limb occlusion and 5‐min reperfusion using a cuff inflator. Further analysis revealed that increased cardiac HIF‐1α expression, increased AKT phosphorylation, and elevated eNOS expression may represent the primary mechanisms underlying RIPC‐induced cardioprotection in this paediatric population [31]. In a T1D rat MIRI model, we further demonstrated that RIPC‐induced protection was mediated by STAT3 phosphorylation at Ser727 [32]; these and other studies position STAT3 as a central hub in the protective signalling network, influencing mitochondrial function, apoptosis and inflammation [33].

### 4.1 Role of STAT3 in Cardioprotective Conditioning

A pivotal feature of STAT3 is its operational independence from other pro‐survival pathways. The conceptual foundation for this independence was established by Lecour, who proposed the SAFE pathway as a STAT3‐dependent cascade distinct from the classical RISK pathway [34]. Genetic evidence confirms that ischemic postconditioning fails to protect STAT3‐deficient mice, whereas pharmacological data show that TNF‐α preconditioning is abrogated by JAK/STAT3 inhibition (AG490) even when AKT signalling remains intact; conversely, protection is preserved when STAT3 is active regardless of the degree of suppression of AKT activity [35]. These findings establish STAT3 as a critical, non‐redundant mediator of cardioprotection, with distinct mitochondrial targets. Mechanistically, mitochondrial STAT3 (mitoSTAT3) is required for optimal ADP‐stimulated respiration and renders mitochondria less susceptible to calcium‐induced mPTP opening through interaction with cyclophilin D [47, 48, 49]. In a porcine model of regional ischemia–reperfusion, Heusch et al. demonstrated that ischemic postconditioning activates mitoSTAT3, preserving complex I respiration and calcium retention capacity—effects abrogated by AG490 in vivo or Stattic in vitro [50]; notably, Stattic itself impairs mitochondrial function via increased ROS production, underscoring the potential risks of indiscriminate STAT3 blockade [51].

STAT3 activation is also a key determinant of the cardioprotective effects of RIPC. The signalling cascade leading to STAT3 activation originates from a neuro‐immune axis: Lieder et al. demonstrated that RIPC in pigs activates a vago‐splenic axis, wherein vagal efferent signalling to the spleen and splenic denervation or splenectomy completely abolishes RIPC‐induced infarct size reduction and the associated increase in myocardial STAT3 phosphorylation. This establishes the vago‐splenic axis as an obligatory upstream component of RIPC signal transduction [52]. In both in vivo porcine and murine hearts treated with plasma from RIPC‐exposed humans, protection was achieved without concurrent activation of the RISK pathway (as evidenced by low or absent phosphorylation of AKT and ERK1/2). However, while porcine and human hearts are protected through STAT3 alone, the transfer of humoral factors to rodent hearts involves both the SAFE and RISK pathways [53]. This species‐specific difference was rigorously demonstrated in a plasma transfer model from RIPC‐treated pigs to isolated rat hearts, where infarct size reduction required both pathways, as blockade of either alone abrogated protection [54]. The humoral transfer of cardioprotection has been confirmed using human plasma in an ex vivo model, where plasma dialysates from healthy volunteers after RIPC were sufficient to confer protection and activate STAT3 in recipient mouse hearts [55]. Human plasma‐dialysates after RIPC similarly conferred protection and STAT3 activation in recipient mouse hearts [56], further confirming the humoral nature of RIPC signalling. In rodents, both pathways are required for infarct size reduction [56, 57]. The core insight is that robust cardioprotection can be achieved through STAT3 activation independent of AKT, challenging the notion that the RISK pathway is always obligatory. In the original species (pigs/humans), the SAFE/STAT3 pathway appears to be predominantly activated, a conclusion directly supported by Gent et al., who reported that IPC in pigs increased STAT3 phosphorylation, while AKT and ERK1/2 phosphorylation occurred irrespective of IPC and that selective STAT3 inhibition alone was sufficient to abolish protection [58].

### 4.2 Extracellular Vesicles as Potential Mediators in Remote Conditioning and STAT3 Signalling Under Diabetic Conditions

In addition to soluble humoral factors including cytokines, chemokines and growth factors, extracellular vehicles (EVs) have recently been recognized as critical carriers of cardioprotective signals between remote organs and the heart, as well as among cardiac cell types such as cardiomyocytes, fibroblasts, endothelial cells and immune cells [59]. EVs contain a rich repertoire of bioactive molecules, including microRNAs (miRNAs), long non‐coding RNAs, proteins and lipids, which can modulate STAT3 activity, inflammatory responses, mitochondrial function and autophagic flux in recipient cells [60]. Remote ischemic conditioning (RIC) induces rapid alterations in circulating EV cargo, including miRNAs known to promote cell survival, attenuate oxidative stress and modulate inflammation and EV‐delivered miRNAs have been proposed as key mediators of RIC‐induced cardioprotection [61]. Importantly, diabetes profoundly alters the quantity, size, cargo profile and functional potency of circulating and cardiac‐derived EVs. Hyperglycemia, insulin resistance and increased oxidative stress drive the enrichment of pro‐inflammatory miRNAs, glycated proteins and ceramides in EVs, while reducing the levels of protective miRNAs such as miR‐126 and miR‐30 [40, 62, 63]. These diabetic EV alterations not only impair the paracrine protective capacity of conditioning stimuli but also render target cardiomyocytes less responsive to STAT3‐dependent prosurvival pathways, thereby contributing to the observed RIC resistance and aggravated myocardial injury in diabetic cardiomyopathy [64, 65]. Although direct evidence linking EV changes to STAT3 dysregulation in diabetic RIC is still emerging, the existing data strongly suggest that EV‐mediated intercellular signalling represents a missing link that warrants further investigation. This perspective is briefly discussed here to complement the humoral mediator framework and to underscore the need for future studies on EV‐based biomarkers and therapeutic strategies in the diabetic heart.

### 4.3 Autonomic Regulation of STAT3 and Mitochondrial Function: Implications for Diabetic Cardiomyopathy

Beyond its role as a conduit for the vago‐splenic anti‐inflammatory axis, the autonomic nervous system—particularly vagal tone—exerts broad regulatory effects on myocardial STAT3 signalling and mitochondrial function that are increasingly recognized as critical determinants of cardioprotective conditioning. Emerging evidence indicates that vagal activity modulates mitochondrial bioenergetics, ROS production and susceptibility to ischemia–reperfusion injury through both direct (neural) and indirect (humoral/inflammatory) pathways. Importantly, recent work by Lionetti et al. demonstrated that right vagus nerve connectivity attenuates cardiomyocyte senescence, reduces inflammatory and complement‐related pathways, improves mitochondrial and metabolic remodelling and preserves cardiac function after injury in a large‐animal model [66].

In the context of diabetes, cardiac autonomic neuropathy (CAN) is a prevalent complication that may contribute to conditioning resistance through mechanisms independent of, or synergistic with intrinsic myocardial signalling defects. Diabetic CAN is characterized by reduced vagal tone and sympathetic overactivity, which collectively impair the autonomic regulation of mitochondrial function and STAT3 phosphorylation [67]. A growing body of work demonstrates that STAT3 exerts its protective effects not only as a nuclear transcription factor but also through a non‐genomic action following its translocation to mitochondria. Within mitochondria, STAT3 enhances complex I activity, curtails excessive reactive oxygen species (ROS) generation and stabilizes the mitochondrial permeability transition pore (mPTP), thereby preserving mitochondrial integrity during ischemic stress [51]. Importantly, vagal tone directly sustains mitochondrial biogenesis and bioenergetic efficiency; loss of vagal input, as seen in diabetes, compromises these regulatory mechanisms and contributes to the premature cardiomyocyte senescence that is a hallmark of diabetic cardiomyopathy [66]. These observations suggest that autonomic–mitochondrial crosstalk represents a previously underappreciated determinant of STAT3‐mediated cardioprotection and that autonomic dysfunction, particularly CAN should be considered as a potential confounder in the translation of conditioning strategies from preclinical models to diabetic patients. Therapeutic interventions aimed at restoring vagal tone, such as vagus nerve stimulation or pharmacological cholinergic agonists, may therefore offer a complementary approach to overcome conditioning resistance in the diabetic heart. Collectively, these observations suggest that the vago‐splenic axis, rather than functioning as an isolated protective mechanism, is best understood as a component of a broader autonomic–mitochondrial STAT3 regulatory network. Future studies should therefore evaluate conditioning strategies in the context of systemic autonomic function, particularly in diabetic patients with CAN, where both central and peripheral vagal inputs may be compromised.

### 4.4 Role of AKT in Cardioprotective Conditioning

The PI3K/AKT pathway is also a well‐established mediator of conditioning strategies. Upon activation by brief ischemia or G‐protein‐coupled receptor agonists, PI3K catalyses the production of phosphatidylinositol (3,4,5)‐trisphosphate (PIP3), which recruits and activates AKT. Once activated, AKT exerts its cardioprotective effects by phosphorylating and inhibiting key proapoptotic proteins such as Bad and GSK‐3β while also stabilizing mitochondrial integrity and suppressing mPTP opening.

Evidence indicates substantial crosstalk between STAT3 and AKT. The inhibition of JAK2/STAT3 abolishes the phosphorylation of AKT and GSK‐3β, resulting in a loss of cardioprotection; conversely, the AKT inhibitor wortmannin inhibits STAT3 phosphorylation, suggesting a bidirectional interaction. During reperfusion, STAT3 is present in both the nucleus and cytoplasm at the embryonic stage, whereas AKT translocates to the nucleus only in more mature neonatal and adult cardiomyocytes. These findings suggest that cross‐talk mechanisms may evolve with cellular maturation. The influence of STAT3 extends to both cytoplasmic AKT and ERK1/2 as well as nuclear targets such as GSK3β. The transient inhibitory effect of STAT3 on nuclear GSK3β during early reoxygenation may be a key determinant of signalling protein transcription and cellular function. Overall, STAT3 may represent a more universally applicable therapeutic target for cardioprotection, although the contribution of AKT must be evaluated within a specific experimental or clinical context [68].

As detailed in Section 3.2, the diabetic myocardium exhibits a ‘STAT3‐deficient’ state driven by multiple converging mechanisms—including upregulated negative regulators, oxidative membrane damage and impaired mitochondrial quality control. This STAT3‐deficient state directly explains why the SAFE pathway, despite being functionally intact in non‐diabetic hearts, fails to be activated by conditioning stimuli in the diabetic setting. Consequently, the protective signals elicited by preconditioning, whether ischemic pre‐, post‐ or remote conditioning—fail to translate into effective defence, leaving the diabetic myocardium refractory to this endogenous cardioprotective strategy. This failure exacerbates apoptosis, increases infarct size and impairs functional recovery, with significant clinical consequences for diabetic patients following acute myocardial infarction (as detailed in Section 2). Therefore, strategies aimed at restoring or enhancing STAT3 signalling are critical and are emerging as promising approaches to alleviate MIRI in the diabetic setting.

## 5 STAT3 As a Key Regulator of Cardiac Protection

In our T2D mouse model of diabetic cardiomyopathy, cardiac dysfunction was closely associated with STAT3 activity; specifically, we observed that reduced Mettl3 expression was restored by exercise, which subsequently activated STAT3 via YB‐1 and ameliorated the cardiomyopathy [69]. This diabetes‐associated impairment of STAT3 cardioprotective signalling is not limited to preclinical models. Owais et al. demonstrated that in myocardial tissues from patients with uncontrolled diabetes undergoing cardiopulmonary bypass (CPB), JAK2 expression and the p‐STAT3/STAT3 ratio were significantly decreased compared with those from nondiabetic patients, accompanied by increased proapoptotic proteins and oxidative stress [70]. However, while this clinical evidence supports the notion of STAT3 pathway desensitization in the diabetic human heart, the CPB‐induced myocardial stress model differs pathophysiologically from acute MIRI. Therefore, direct extrapolation of the preclinical MIRI findings to human acute coronary syndromes warrants cautious interpretation, and future clinical investigations specifically targeting STAT3 alterations in diabetic patients with MIRI are clearly needed.

The STAT family comprises seven cytoplasmic transcription factors—STAT1, STAT2, STAT3, STAT4, STAT5A, STAT5B and STAT6—that relay signals from cell surface receptors to the nucleus. These STAT proteins are ubiquitously expressed throughout the heart and within individual cardiomyocytes [71]. They are activated by a diverse array of cytokines, growth factors and hormones and classically mediate various cellular processes, including proliferation, differentiation and immune responses [72]. Within this family, STAT3 has emerged as a particularly critical and multifaceted regulator of cardiac homeostasis and stress adaptation [73].

STAT3 activation is primarily triggered by the binding of ligands, such as interleukin‐6 (IL‐6), leukaemia inhibitory factor (LIF) [74] and cardiotrophin‐1, to their respective gp130 receptor complexes [53, 75]. This binding triggers the recruitment and activation of Janus kinases (JAKs), which then phosphorylate specific tyrosine residues, notably Tyr705 of STAT3. Tyrosine phosphorylation induces STAT3 dimerization, nuclear translocation and binding to specific promoter sequences, thereby regulating the transcription of a broad range of target genes [76]. In the heart, the STAT3 transcriptional program is highly cardioprotective: it increases the expression of antiapoptotic proteins, such as Bcl‐2 and Bcl‐xL, as well as potent antioxidants, such as manganese superoxide dismutase (MnSOD) [77]. It also regulates the expression of genes involved in angiogenesis [78] and metabolic adaptation [79].

Notably, STAT5, a closely related family member, also plays an important role in the heart. STAT5 is a key promoter of cardiomyocyte survival and hypertrophy and is activated by hormones such as growth hormone and prolactin, as well as certain cytokines [80]. It shares some protective functions with STAT3, including the induction of Bcl‐xL expression. While STAT3 is generally considered the predominant STAT family member involved in conditioning‐induced cardioprotection [81], emerging evidence suggests a more nuanced picture. In a clinical study by Thielmann and colleagues, compared with the control procedure, three cycles of 5‐min arm ischemia followed by 5‐min reperfusion, administered prior to coronary artery bypass surgery, significantly reduced troponin I levels. Notably, STAT5 protein expression was increased in RIPC‐treated patients compared with controls, whereas no change in STAT3 expression was detected [82]. These findings suggest that different STAT family members may predominate in different clinical contexts. Nevertheless, when STAT3 is activated, it can orchestrate protection independently of the PI3K/Akt axis, providing an essential and parallel survival pathway.

## 6 Relationship of STAT3 With Mitochondrial Metabolism

As established in Section 3.2, diabetes‐induced oxidative stress and mitochondrial dysfunction converge to suppress STAT3 activation and compromise mitochondrial quality control. Beyond this upstream regulatory framework, a distinct pool of STAT3 resides within the mitochondrial matrix (mitoSTAT3) and exerts direct, non‐transcriptional control over organelle function [50]. Here, we focus specifically on the effector mechanisms of mitoSTAT3—how it modulates electron transport chain complex I/II activity, regulates ADP‐stimulated respiration, restricts ROS production through efficient electron flow and maintains calcium retention capacity to inhibit mPTP opening [83, 84]. These effector functions are directly relevant to cardioprotection, as mitoSTAT3 deficiency in diabetes (discussed in Section 3.2) would deprive the diabetic heart of this intrinsic mitochondrial defence.

This mitochondrial homeostatic network is profoundly disrupted in diabetes. A defining feature of diabetic mitochondria is metabolic inflexibility [12], the impaired ability to switch between fuel substrates. This metabolic overload results in incomplete oxygen reduction and excessive ROS and oxidative stress, which damage mitochondrial DNA, lipids and proteins, establishing a self‐perpetuating cycle of injury [85]. This ROS overproduction is accompanied by reduced antioxidant defences and diminished mitochondrial biogenesis, driven by downregulation of key regulators such as PGC‐1α. Consequently, the ETC becomes less efficient, the mitochondrial membrane potential decreases, ATP synthesis decreases and mPTP opening is promoted—a key event in apoptotic cell death. This collective mitochondrial failure directly underlies the diabetic complications observed in peripheral nerves, the retina and the cardiovascular system [86]. This collective mitochondrial failure, compounded by the documented reduction in cardiac STAT3 levels, directly compromises both mitochondrial bioenergetics and ROS restraint, rendering the myocardium particularly vulnerable to MIRI and refractory to conditioning stimuli.

Equally importantly, mitoSTAT3 restricts ROS production by ensuring efficient electron flow through the ETC, thereby minimizing electron leakage and subsequent superoxide formation [50]. During MIRI, when a burst of mitochondrial ROS is the primary driver of damage, this ROS‐restraining function of mitoSTAT3 is of paramount importance. MitoSTAT3 also helps maintain the mitochondrial membrane potential (ΔΨm) and inhibits mPTP opening through interactions with cyclophilin D [51, 87]. Boengler et al. provided the first direct evidence that mitochondrial STAT3 deficiency impairs respiration and sensitizes mitochondria to calcium‐induced mPTP opening, establishing a mechanistic link between mitoSTAT3 and cardioprotection [87].

The molecular mechanisms by which heart ischemia conditioning disrupts STAT3‐mediated cardioprotection, including defective upstream JAK/STAT activation, impaired mitochondrial STAT3 signalling, enhanced oxidative stress and altered metabolic signalling, are illustrated in Figure 2. The posttranslational modifications shown in Figure 2 are discussed in detail in Section 6.1.

**FIGURE 2:**
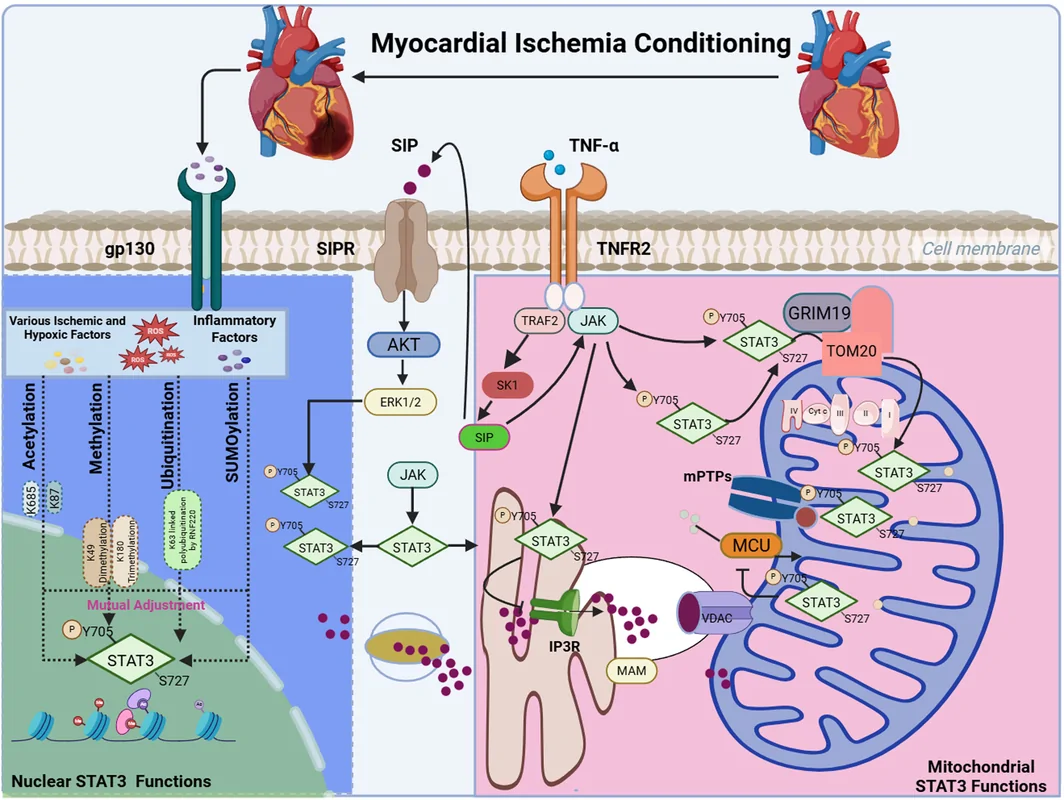
Myocardial ischemia–reperfusion injury (MIRI) triggers the release of various ischemic/hypoxic factors, ROS and inflammatory cytokines (e.g., TNF‐α). These extracellular stimuli activate plasma membrane receptors (such as TNFR2, gp130 and SIPR) to initiate downstream signalling cascades, including the JAK/STAT3, AKT and ERK1/2 pathways. Upon activation, STAT3 undergoes phosphorylation at Tyr705 and Ser727, Additionally, STAT3 is subject to putative post‐translational modifications within the nucleus (depicted by dotted lines), such as acetylation at Lys685, methylation at Lys49 and Lys180, ubiquitination and SUMOylation, which are thought to fine‐tune its transcriptional activity. Furthermore, STAT3 localizes to the mitochondria, where it interacts with proteins such as GRIM19 and TOM20 at the outer membrane and modulates the function of the mitochondrial permeability transition pore (mPTPs) and MCU at the inner membrane (via interactions with VDAC and IP3R at the mitochondria‐associated membranes, MAM). These mitochondrial actions regulate oxidative phosphorylation, ATP production, mtROS generation, mitochondrial membrane potential, apoptosis and mitophagy, collectively contributing to the cardioprotective effects of ischemic conditioning. (Figure created with BioRender.com).

### 6.1 STAT3 Post‐Translational Modifications: Beyond Phosphorylation

Beyond the canonical Tyr705 and Ser727 phosphorylation, STAT3 activity is finely tuned by a network of post‐translational modifications (PTMs) that remain largely unexplored in diabetic MIRI. Acetylation at K685, which is catalysed by p300/CBP, increases STAT3 transcriptional activity and dimer stability [88], whereas acetylation at K87 facilitates mitochondrial translocation. Methylation at K49 (dimethylation) and K180 (trimethylation) modulates STAT3 transcriptional output and subcellular partitioning. Ubiquitination such as K63‐linked polyubiquitination by RNF220 stabilizes STAT3, whereas SUMOylation regulates its interaction with partner proteins [89]. Given that diabetes features oxidative stress and chronic inflammation, both are potent inducers of PTM‐regulating enzymes such as p300 and EZH2, it is highly plausible that aberrant PTMs contribute to the ‘STAT3‐deficient’ state observed in the diabetic heart [90]. Indeed, STAT3 deficiency has been shown to downregulate MnSOD expression, leading to oxidative stress and subsequent Cathepsin D‐mediated cleavage of prolactin, a process that contributes to peripartum cardiomyopathy [91]. These findings underscore that STAT3 deficiency not only compromises acute stress responses but also serves as a primary driver of cardiomyopathy under specific metabolic and hormonal conditions. However, to date, no study has systematically examined STAT3 acetylation, methylation or SUMOylation in the context of diabetic MIRI. Deciphering the STAT3 PTM landscape under diabetic conditions may uncover novel therapeutic entry points beyond traditional phospho‐STAT3 targeting and should be prioritized in future mechanistic investigations. Consistent with this endeavour, in Figure 2 we have distinguished established modifications (Tyr705 and Ser727 phosphorylation, denoted by solid lines) from speculative ones (acetylation, methylation, SUMOylation and ubiquitination, denoted by dashed lines) to clearly convey that while the latter are conceptually attractive candidates, their direct involvement in diabetic MIRI remains unproven and warrants systematic investigation.

## 7 Context‐Dependent Regulation of STAT3 in the Diabetic Heart

Although reduced STAT3 activation has frequently been reported in diabetic hearts, the literature is heterogeneous and at times contradictory. Differences in diabetes model, disease duration, cellular compartment and species can all influence the direction and magnitude of STAT3 regulation. Below, we summarize these sources of heterogeneity and propose plausible explanatory factors.

### 7.1 Diabetes Model: Type 1 Versus Type 2

Most preclinical studies have used either streptozotocin (STZ)‐induced type 1 diabetes (T1D) or genetic/diet‐induced type 2 diabetes (T2D) models such as db/db, ob/ob or high‐fat diet (HFD) feeding. In STZ‐T1D models, absolute insulin deficiency and severe hyperglycemia are typically associated with reduced total STAT3 expression and decreased phosphorylation at Tyr705 and Ser727, consistent with impaired JAK2/STAT3 signalling and enhanced activity of negative regulators such as FOXO1, PHLPP1 and protein tyrosine phosphatases [12, 36, 37, 38, 39]. By contrast, in T2D models characterized by insulin resistance and hyperinsulinemia, STAT3 regulation appears more variable: some studies report attenuated STAT3 phosphorylation and transcriptional activity, whereas others describe preserved or even enhanced STAT3 activation in early stages, potentially reflecting compensatory responses to chronic metabolic stress and inflammation. These discrepancies may arise from differences in the severity of insulin resistance, adiposity, systemic inflammation and the balance between pro‐survival cytokines such as IL‐6, LIF and negative feedback pathways, including SOCS3, PTP1B [92, 93].

### 7.2 Disease Stage: Early Compensatory Activation Versus Chronic Desensitization

The temporal dimension of diabetes is another critical determinant of STAT3 behaviour. In early or acute phases of hyperglycemia and metabolic stress, several models have shown transient upregulation or hyperphosphorylation of STAT3, particularly at Tyr705, which may represent a compensatory attempt to maintain cardiomyocyte survival and antioxidant defences. However, with prolonged hyperglycemia, chronic oxidative stress and sustained inflammation, this compensatory activation often gives way to desensitization or suppression of STAT3 signalling, characterized by reduced phosphorylation, increased dephosphorylation by PTPs and enhanced expression of inhibitory proteins such as SOCS3 [92]. This time‐dependent shift may explain why some studies that sampled tissue at early time points report increased STAT3 activation, whereas others that examined chronic diabetes consistently observe STAT3 downregulation and impaired SAFE signalling [94].

### 7.3 Cellular Compartment: Nuclear Versus Mitochondrial STAT3

STAT3 exerts distinct functions in the nucleus and mitochondria, mediated largely by phosphorylation at Tyr705 and Ser727, respectively. In many diabetic models, nuclear STAT3 activity (p‐Tyr705) is reduced, leading to decreased transcription of anti‐apoptotic and antioxidant genes such as Bcl‐2, Bcl‐xL and MnSOD [76]. Mitochondrial STAT3 (mitoSTAT3), which modulates electron transport chain activity, ROS production and mPTP susceptibility, may be differentially affected [68]. Some studies in diabetic hearts report parallel reductions in both p‐Tyr705 and p‐Ser727, suggesting global STAT3 impairment, whereas others indicate that Ser727 phosphorylation and mitoSTAT3 localization may be relatively preserved or regulated by distinct mechanisms, such as acetylation or interaction with cyclophilin D. These compartment‐specific patterns underscore the importance of distinguishing total STAT3 from phospho‐isoforms and from nuclear versus mitochondrial pools when interpreting apparently contradictory findings [95].

### 7.4 Species Differences: Rodents Versus Large Animals and Humans

Most mechanistic insights into STAT3 in diabetic MIRI derive from rodent models, but translational relevance to large animals and humans remains uncertain. Rodent hearts exhibit higher basal respiratory rates, greater reliance on carbohydrate oxidation and different calcium handling compared with human myocardium, which may influence both the magnitude of STAT3 activation and its functional consequences during ischemia–reperfusion [70, 96]. Furthermore, interspecies differences in substrate utilization, redox balance and mitochondrial susceptibility to mPTP opening could modulate the extent to which mitoSTAT3 confers protection. Clinical data from diabetic patients undergoing cardiopulmonary bypass show reduced JAK2 expression and lower p‐STAT3/STAT3 ratios compared with non‐diabetic controls, supporting the notion of STAT3 pathway desensitization in the human diabetic heart [70]. However, direct comparisons across species under standardized diabetic and ischemia–reperfusion conditions are lacking and it remains possible that the net effect of diabetes on STAT3‐mediated cardioprotection differs quantitatively, if not qualitatively, between rodents and humans.

Collectively, these four axes—diabetes model, disease stage, cellular compartment and species provide a framework for reconciling seemingly contradictory reports on STAT3 in diabetic cardiomyopathy. Rather than reflecting true inconsistency, the literature likely captures a dynamic and context‐dependent regulatory landscape in which STAT3 can be upregulated, downregulated or unchanged depending on the specific experimental or clinical setting. Recognizing this heterogeneity is essential for designing future studies that systematically compare different models, time courses and cell types and for developing STAT3‐targeted interventions that are tailored to specific diabetic subpopulations.

## 8 Challenges and Future Considerations

Ischemic conditioning, particularly remote ischemic preconditioning (RIPC), has successfully transitioned from experimental animal models to clinical applications, demonstrating clear cardioprotective efficacy against MIRI. However, one persistent challenge in clinical translation is the lack of real‐time indicators to assess whether a conditioning stimulus has indeed engaged its protective targets. In this context, studies in pigs have shown that attenuation of ST‐segment elevation during coronary occlusion may serve as an online reflection of cardioprotection by RIPC [97]. Nevertheless, whether such electrocardiographic surrogates are applicable in the diabetic heart and where baseline ST‐segment changes and autonomic neuropathy may confound interpretation remain to be determined.

Beyond this monitoring challenge, targeting STAT3 to restore cardioprotection in the diabetic heart faces substantial biological obstacles. A primary obstacle is the dual nature of STAT3 signalling, as while acute STAT3 activation is protective in conditioning paradigms, chronic sustained STAT3 activation promotes pathological cardiac hypertrophy and inflammation. Thus, STAT3 activation must be precisely calibrated to elicit transient protection without triggering adverse long‐term effects. Moreover, reduced STAT3 expression and phosphorylation in the diabetic myocardium may render conditioning stimuli ineffective in many, but not all, models, positioning STAT3 deficiency as a potential contributor to cardioprotection resistance in diabetes. Beyond these well‐recognized obstacles, several less‐defined factors may further modulate STAT3‐mediated cardioprotection in the diabetic heart.

The role of STAT5, which shares some functional overlap with that of STAT3 in promoting cell survival, remains unclear. Future investigations should determine whether STAT5 activation can partially compensate for STAT3 deficiency in diabetes or whether it is similarly impaired. Additionally, sex differences in STAT3‐mediated cardioprotection have been increasingly recognized; diabetic women with STEMI exhibit worse outcomes than men do and preclinical evidence suggests that oestrogen signalling enhances STAT3 activation, while testosterone may suppress it. These observations underscore the need to include both sexes in future mechanistic and therapeutic studies.

Looking forward, research should prioritize several key areas. First, safe and effective pharmacological or genetic approaches must be developed to selectively increase STAT3 activation prior to planned ischemic events or at infarction onset, particularly in patients with diabetes, potentially via STAT3 agonists or agents that modulate upstream regulators such as IL‐6 and/or gp130. Second, exploring the synergistic potential of combining STAT3 activation with other protective modalities, such as glucagon‐like peptide‐1 receptor agonists or sodium‐glucose cotransporter 2 inhibitors, represents a compelling therapeutic avenue, as these agents may positively influence survival pathways. Third, a deeper understanding of the interaction between STAT3 and STAT5 and their respective contributions to the diabetic phenotype is essential. Fourth, elucidating the precise molecular mechanisms underlying STAT3 dysfunction in diabetes patients, including the PTM landscape discussed above, will be critical for developing novel clinical interventions. Looking forward, we emphasize that the net effect of diabetes on STAT3‐mediated cardioprotection is likely context‐dependent. Future studies should systematically compare different diabetes models (STZ‐induced type 1 vs. genetic or high‐fat diet‐induced type 2), disease time courses (early compensatory vs. chronic desensitization phases) and cell types (cardiomyocyte vs. fibroblast vs. immune cell) to better define the conditions under which STAT3‐targeted strategies might be beneficial or ineffective. Such systematic comparisons are essential to resolve current contradictions in the literature and to guide the rational design of STAT3‐targeted cardioprotective interventions for specific diabetic subpopulations. Finally, we acknowledge that autonomic dysfunction, particularly CAN in diabetes, should be considered as a potential confounder in the translation of conditioning strategies from preclinical models to clinical practice. As discussed in the autonomic regulation section, impaired vagal tone compromises mitochondrial STAT3 signalling and contributes to cardiomyocyte senescence. Therefore, interventions targeting autonomic function, such as vagus nerve stimulation or pharmacological cholinergic agonists, may offer a complementary strategy to restore STAT3‐dependent cardioprotection in diabetic patients, particularly those with CAN.

## 9 Conclusions

In healthy myocardium, STAT3 functions as a bifunctional guardian: in the nucleus, it drives the transcription of anti‐apoptotic and antioxidant genes, including Bcl‐2, Bcl‐xL and MnSOD; in the mitochondria, it preserves electron transport chain efficiency, maintains membrane potential and restrains mPTP opening. These dual actions converge to render cardiomyocytes resilient to the oxidative, inflammatory and bioenergetic insults that define reperfusion injury. Importantly, the integrity of this STAT3‐mediated protective architecture depends not only on intrinsic myocardial signalling but also on intact autonomic inputs. Autonomic dysfunction, particularly CAN in diabetes, may therefore act as a confounding factor that limits the efficacy of conditioning strategies in diabetic patients.

However, the net effect of diabetes on STAT3‐mediated cardioprotection is likely context‐dependent, rather than a uniform suppression. While chronic hyperglycemia, insulin resistance, oxidative stress and low‐grade inflammation often suppress STAT3 expression and may reduce its phosphorylation at Tyr705 and Ser727 in many experimental settings, the direction and magnitude of these changes vary substantially across diabetes models (STZ‐induced type 1 vs. genetic or high‐fat diet‐induced type 2), disease stages (early compensatory activation vs. chronic desensitization) and cellular compartments (nuclear vs. mitochondrial STAT3). This context‐dependent STAT3 dysregulation—ranging from early compensatory hyperphosphorylation to late‐stage desensitization—uncouples the SAFE pathway from conditioning stimuli under certain conditions, rendering cardioprotective strategies ineffective in specific diabetic contexts. The downstream consequences include larger infarct sizes, worse ventricular function, heightened apoptosis and poorer clinical outcomes following acute myocardial infarction in affected patient subsets. Achieving the goal of restoring STAT3‐mediated cardioprotection will require not only a deeper mechanistic understanding but also a paradigm shift in trial design that stratifies patients by diabetic subtype, sex, disease duration and STAT3 functional status, ultimately translating STAT3‐targeted cardioprotection from the bench to the bedside.

## 10 Limitations

Several limitations must be acknowledged. First, most of the mechanistic studies reviewed herein were conducted in rodent models of T1D or T2D, and the translational relevance of these findings to the human diabetic heart remains incompletely established. A particularly critical caveat in this regard is the substantial interspecies heterogeneity in mitochondrial physiology. While the RISK/SAFE pathways and mitoSTAT3 have been extensively characterized in rodent models, the mitochondrial phenotype of rodents differs profoundly from that of large animals (e.g., pigs) and humans. Specifically, rodent mitochondria exhibit higher basal respiratory rates, greater reliance on carbohydrate oxidation, lower ROS production per unit of oxygen consumed and higher calcium retention capacity compared with human mitochondria, which display higher fatty acid oxidation rates and greater susceptibility to calcium‐induced mPTP opening [98]. These differences have direct consequences for mitoSTAT3 signalling, as STAT3 resides within the mitochondrial matrix and interacts with complex I and II of the electron transport chain, modulating the ROS burst and mPTP threshold during ischemia–reperfusion. Given that ROS generation and calcium dynamics are markedly species‐dependent, the magnitude of mitoSTAT3 phosphorylation at Ser727 and its protective effects on mitochondrial integrity may be overestimated in rodent models. Moreover, interspecies variations in substrate utilization including fatty acids and glucose, may influence the extent to which mitoSTAT3 modulates electron transport, ROS generation and mPTP susceptibility during ischemia–reperfusion. Consequently, cardioprotective conditioning strategies that rely on mitoSTAT3 activation may yield robust effects in rodents but show attenuated or even absent efficacy in human myocardium. This translational gap underscores the imperative for validating mitoSTAT3‐centric interventions in clinically relevant large‐animal models of ischemia–reperfusion injury, as recently advocated, before progressing to human trials [51, 68, 87, 98]. Second, the changes in STAT3 expression and phosphorylation reported across diabetic MIRI studies are paradoxical and contradictory. Some investigations have reported reduced STAT3 levels and phosphorylation in diabetic hearts, whereas others have demonstrated context‐dependent upregulation. This heterogeneity likely reflects differences in the type of diabetes model (T1D or T2D), disease duration, ischemia–reperfusion protocol and timing of tissue sampling. Standardization of experimental designs is essential for resolving these discrepancies. Finally, the therapeutic dual nature of STAT3 imposes important constraints on translational strategies: while transient acute STAT3 activation is cardioprotective, chronic overactivation is associated with pathological hypertrophy, fibrosis and maladaptive remodelling, as noted in Section 7.

## Author Contributions

**Chunyan Wang:** funding acquisition, writing – original draft, methodology. **Wangli Tian:** methodology, software. **Saman Saedi:** software, methodology, writing – review and editing. **Hengzhen Cui:** investigation, formal analysis. **Danyong Liu:** formal analysis, resources. **Qinjun Chu:** writing – review and editing. **Dongwei Yang:** investigation, data curation. **Zhengyuan Xia:** writing – review and editing.

## Funding

This study was supported by grants from the Shenzhen Science and Technology Innovation Committee (grant nos. JCYJ20240813115006008 and JCYJ20230807120211022) and the National Natural Science Foundation of China (grant no. NSFC 82270306).

## Disclosure

Declaration of the Use of Generative AI and AI‐Assisted Technologies in the Writing Process: Except for checking grammar and spelling, no AI‐assisted technology was used in the preparation of this article.

## Conflicts of Interest

The authors declare no conflicts of interest.

## Data Availability

No data were created or analysed in this study. Data sharing is not applicable to this article.

## References

[1] L. Zhong , Z. Cheng , Y. Zhang , et al., “Cardiomyocyte‐Derived GPX4 Stabilizes BNIP3 to Facilitate Mitophagy and Mitigate Myocardial Ischemia/Reperfusion Injury,” Nature Communications 17 (2026): 4578. 10.1038/s41467-026-71232-2 PMC13194750 41904174

[2] G. Heusch , “Myocardial Ischemia/Reperfusion: Translational Pathophysiology of Ischemic Heart Disease,” Medicus 5 (2024): 10–31. 10.1016/j.medj.2023.12.007 38218174

[3] J. He , D. Liu , L. Zhao , et al., “Myocardial Ischemia/Reperfusion Injury: Mechanisms of Injury and Implications for Management (Review),” Experimental and Therapeutic Medicine 23 (2022): 430. 10.3892/etm.2022.11357 PMC9121204 35607376

[4] G. Heusch , “Myocardial Ischaemia‐Reperfusion Injury and Cardioprotection in Perspective,” Nature Reviews. Cardiology 17 (2020): 773–789. 10.1038/s41569-020-0403-y 32620851

[5] P. Ferdinandy , I. Andreadou , G. F. Baxter , et al., “Interaction of Cardiovascular Nonmodifiable Risk Factors, Comorbidities and Comedications With Ischemia/Reperfusion Injury and Cardioprotection by Pharmacological Treatments and Ischemic Conditioning,” Pharmacological Reviews 75 (2023): 159–216. 10.1124/pharmrev.121.000348 PMC9832381 36753049

[6] J. Xue , J. Zhuang , X. Wang , et al., “Mechanisms and Therapeutic Strategies for Myocardial Ischemia‐Reperfusion Injury in Diabetic States,” ACS Pharmacology & Translational Science 7 (2024): 3691–3717. 10.1021/acsptsci.4c00272 PMC11651189 39698288

[7] S. Kant and J. Feng , “Protein Kinase C and Endothelial Dysfunction in Select Vascular Diseases,” Frontiers in Cardiovascular Medicine 12 (2025): 1618343. 10.3389/fcvm.2025.1618343 PMC12414950 40926896

[8] H. L. Li , G. Y. H. Lip , Q. Feng , et al., “Sodium‐Glucose Cotransporter 2 Inhibitors (SGLT2i) and Cardiac Arrhythmias: A Systematic Review and Meta‐Analysis,” Cardiovascular Diabetology 20 (2021): 100. 10.1186/s12933-021-01293-8 PMC8106208 33962654

[9] H. Huang , Y. Ruan , C. Li , et al., “Hypoxia Microenvironment Preconditioning Attenuated Myocardial Ischemia‐Reperfusion Injury via Stc1‐Mediating Cardiomyocyte Self‐Protection and Neutrophil Polarization,” Advanced Science 12 (2025): e2411880. 10.1002/advs.202411880 PMC11809403 39676707

[10] A. Lopez‐de‐Andres , R. Jimenez‐Garcia , V. Hernandez‐Barrera , et al., “Are There Sex Differences in the Effect of Type 2 Diabetes in the Incidence and Outcomes of Myocardial Infarction? A Matched‐Pair Analysis Using Hospital Discharge Data,” Cardiovascular Diabetology 20 (2021): 81. 10.1186/s12933-021-01273-y PMC8063379 33888124

[11] C. Torregroza , L. Gnaegy , A. Raupach , et al., “Influence of Hyperglycemia and Diabetes on Cardioprotection by Humoral Factors Released After Remote Ischemic Preconditioning (RIPC),” International Journal of Molecular Sciences 22 (2021): 8880. 10.3390/ijms22168880 PMC8396298 34445586

[12] L. Zhou , W. Su , Y. Wang , Y. Zhang , Z. Xia , and S. Lei , “FOXO1 Reduces STAT3 Activation and Causes Impaired Mitochondrial Quality Control in Diabetic Cardiomyopathy,” Diabetes, Obesity & Metabolism 26 (2024): 732–744. 10.1111/dom.15369 37961034

[13] L. Wang , D. Li , F. Yao , et al., “Serpina3k Lactylation Protects From Cardiac Ischemia Reperfusion Injury,” Nature Communications 16 (2025): 1012. 10.1038/s41467-024-55589-w PMC11760901 39856050

[14] X. Cheng , S. Liu , Y. Zhang , et al., “Remote Activation of Spinal TRPV1 by Magnetic Nanocubes Confers Cardioprotection Against Myocardial Ischemia‐Reperfusion Injury,” Advanced Science (Weinheim) 13 (2025): e20852. 10.1002/advs.202520852 PMC12931263 41387112

[15] C. Wang , L. Zhu , W. Yuan , et al., “Diabetes Aggravates Myocardial Ischaemia Reperfusion Injury via Activating Nox2‐Related Programmed Cell Death in an AMPK‐Dependent Manner,” Journal of Cellular and Molecular Medicine 24 (2020): 6670–6679. 10.1111/jcmm.15318 PMC7299688 32351005

[16] Y. Abdallah , S. A. Kasseckert , W. Iraqi , et al., “Interplay Between Ca2+ Cycling and Mitochondrial Permeability Transition Pores Promotes Reperfusion‐Induced Injury of Cardiac Myocytes,” Journal of Cellular and Molecular Medicine 15 (2011): 2478–2485. 10.1111/j.1582-4934.2010.01249.x PMC3822958 21199327

[17] G. Kung , K. Konstantinidis , and R. N. Kitsis , “Programmed Necrosis, Not Apoptosis, in the Heart,” Circulation Research 108 (2011): 1017–1036, 10.1161/CIRCRESAHA.110.225730. PMC12947135 21493924

[18] T. Zhang , Y. Zhang , M. Cui , et al., “CaMKII Is a RIP3 Substrate Mediating Ischemia‐ and Oxidative Stress‐Induced Myocardial Necroptosis,” Nature Medicine 22 (2016): 175–182. 10.1038/nm.4017 26726877

[19] A. P. Halestrap , “A Pore Way to Die: The Role of Mitochondria in Reperfusion Injury and Cardioprotection,” Biochemical Society Transactions 38 (2010): 841–860, 10.1042/BST0380841. 20658967

[20] F. A. Zouein and G. W. Booz , “Targeting Mitochondria to Protect the Heart: A Matter of Balance?,” Clinical Science (London, England) 139 (2025): 1591–1596. 10.1042/CS20258287 PMC12751048 41359793

[21] H. R. Yun , M. K. Singh , S. Han , et al., “Roles of Autophagy and Oxidative Stress in Cardiovascular Disease,” Antioxidants (Basel) 14 (2025): 14. 10.3390/antiox14101263 PMC12561683 41154572

[22] J. S. Hawi , K. L. Skeffington , M. Young , M. Caputo , R. Ascione , and M. S. Suleiman , “Effect of High‐Fat Diet on Cardiac Metabolites and Implications for Vulnerability to Ischemia and Reperfusion Injury,” Cells 14 (2025): 14. 10.3390/cells14171329 PMC12428587 40940740

[23] S. H. Hur , K. B. Won , I. C. Kim , et al., “Comparison of 2‐Year Clinical Outcomes Between Diabetic Versus Nondiabetic Patients With Acute Myocardial Infarction After 1‐Month Stabilization: Analysis of the Prospective Registry of DIAMOND (DIabetic Acute Myocardial infarctiON Disease) in Korea: An Observational Registry Study,” Medicine (Baltimore) 95 (2016): e3882. 10.1097/MD.0000000000003882 PMC4998313 27336875

[24] A. Al‐Kouh and F. Babiker , “Nitric Oxide/Glucose Transporter Type 4 Pathway Mediates Cardioprotection Against Ischemia/Reperfusion Injury Under Hyperglycemic and Diabetic Conditions in Rats,” Journal of Vascular Research 61 (2024): 179–196. 10.1159/000539461 38952123

[25] P. Sethupathi , A. Matetic , V. Bang , et al., “Association of Diabetes Mellitus and Its Types With In‐Hospital Management and Outcomes of Patients With Acute Myocardial Infarction,” Cardiovascular Revascularization Medicine 52 (2023): 16–22. 10.1016/j.carrev.2023.02.008 36854639

[26] T. A. Fairbairn , D. M. Meads , A. N. Mather , et al., “Serial Change in Health‐Related Quality of Life Over 1 Year After Transcatheter Aortic Valve Implantation: Predictors of Health Outcomes,” Journal of the American College of Cardiology 59 (2012): 1672–1680. 10.1016/j.jacc.2012.01.035 22554597

[27] A. K. Gitt , R. Schiele , H. Wienbergen , et al., “Intensive Treatment of Coronary Artery Disease in Diabetic Patients in Clinical Practice: Results of the MITRA Study,” Acta Diabetologica 40, no. Suppl 2 (2003): S343–S347. 10.1007/s00592-003-0117-8 14704866

[28] R. Sedhom , N. Murthy , M. Megaly , et al., “Characteristics, Management, and Outcomes of Diabetes Subtypes in Patients With Cardiogenic Shock: A Nationwide Analysis,” JACC: Advances 5 (2026): 102766. 10.1016/j.jacadv.2026.102766 PMC13140053 42202375

[29] V. Subramanian , K. V. Patel , J. M. Suh , et al., “Screening and Treatment Using a Risk‐Based Approach With NT‐proBNP Guidance in Diabetes Mellitus: The STRONG‐DM Pilot Study,” JACC Heart Failure 14 (2026): 103153. 10.1016/j.jchf.2026.103153 42233896

[30] L. Teng , E. Bennett , and C. Cai , “Preconditioning c‐Kit‐Positive Human Cardiac Stem Cells With a Nitric Oxide Donor Enhances Cell Survival Through Activation of Survival Signaling Pathways,” Journal of Biological Chemistry 291 (2016): 9733–9747. 10.1074/jbc.M115.687806 PMC4850310 26940876

[31] Q. Wu , T. Wang , S. Chen , et al., “Cardiac Protective Effects of Remote Ischaemic Preconditioning in Children Undergoing Tetralogy of Fallot Repair Surgery: A Randomized Controlled Trial,” European Heart Journal 39 (2018): 1028–1037. 10.1093/eurheartj/ehx030 PMC6018784 28329231

[32] C. Wang , H. Li , S. Wang , et al., “Repeated Non‐Invasive Limb Ischemic Preconditioning Confers Cardioprotection Through PKC‐Ԑ/STAT3 Signaling in Diabetic Rats,” Cellular Physiology and Biochemistry 45 (2018): 2107–2121. 10.1159/000488047 29533954

[33] A. Haghikia , B. Stapel , M. Hoch , and D. Hilfiker‐Kleiner , “STAT3 and Cardiac Remodeling,” Heart Failure Reviews 16 (2011): 35–47. 10.1007/s10741-010-9170-x 20407820

[34] S. Lecour , “Activation of the Protective Survivor Activating Factor Enhancement (SAFE) Pathway Against Reperfusion Injury: Does It Go Beyond the RISK Pathway?,” Journal of Molecular and Cellular Cardiology 47 (2009): 32–40. 10.1016/j.yjmcc.2009.03.019 19344728

[35] S. Lecour , N. Suleman , G. A. Deuchar , et al., “Pharmacological Preconditioning With Tumor Necrosis Factor‐Alpha Activates Signal Transducer and Activator of Transcription‐3 at Reperfusion Without Involving Classic Prosurvival Kinases (Akt and Extracellular Signal‐Regulated Kinase),” Circulation 112 (2005): 3911–3918. 10.1161/CIRCULATIONAHA.105.581058 16344382

[36] F. Andreozzi , C. Procopio , A. Greco , et al., “Increased Levels of the Akt‐Specific Phosphatase PH Domain Leucine‐Rich Repeat Protein Phosphatase (PHLPP)‐1 in Obese Participants Are Associated With Insulin Resistance,” Diabetologia 54 (2011): 1879–1887. 10.1007/s00125-011-2116-6 21461637

[37] S. Gao , Y. Qiu , Y. Meng , et al., “Blockage of PHLPP1 Protects Against Myocardial Ischemia/Reperfusion Injury in Diabetic Mice via Activation of STAT3 Signaling,” Journal of Bioenergetics and Biomembranes 55 (2023): 325–339. 10.1007/s10863-023-09977-4 37584737

[38] A. Fukushima , K. Loh , S. Galic , et al., “T‐Cell Protein Tyrosine Phosphatase Attenuates STAT3 and Insulin Signaling in the Liver to Regulate Gluconeogenesis,” Diabetes 59 (2010): 1906–1914. 10.2337/db09-1365 PMC2911070 20484139

[39] L. Grant , K. D. Shearer , A. Czopek , et al., “Myeloid‐Cell Protein Tyrosine Phosphatase‐1B Deficiency in Mice Protects Against High‐Fat Diet and Lipopolysaccharide‐Induced Inflammation, Hyperinsulinemia, and Endotoxemia Through an IL‐10 STAT3‐Dependent Mechanism,” Diabetes 63 (2014): 456–470. 10.2337/db13-0885 24186864

[40] X. Huang , J. Guo , A. Ning , N. Zhang , and Y. Sun , “BAG3 Promotes Proliferation and Migration of Arterial Smooth Muscle Cells by Regulating STAT3 Phosphorylation in Diabetic Vascular Remodeling,” Cardiovascular Diabetology 23 (2024): 140. 10.1186/s12933-024-02216-z PMC11046803 38664681

[41] M. Manojlovic , S. Zafirovic , D. Tomic Naglic , et al., “Mitochondrial Dysfunction, Reactive Oxygen Species, and Diabetes Mellitus—A Triangular Relationship: A Review,” Biomolecules and Biomedicine 26 (2025): 547–558. 10.17305/bb.2025.13145 PMC12710611 41128021

[42] S. Lei , W. Su , Z. Y. Xia , et al., “Hyperglycemia‐Induced Oxidative Stress Abrogates Remifentanil Preconditioning‐Mediated Cardioprotection in Diabetic Rats by Impairing Caveolin‐3‐Modulated PI3K/Akt and JAK2/STAT3 Signaling,” Oxidative Medicine and Cellular Longevity 2019 (2019): 9836302. 10.1155/2019/9836302 PMC6748204 31583053

[43] G. Heusch , I. Andreadou , R. Bell , et al., “Health Position Paper and Redox Perspectives on Reactive Oxygen Species as Signals and Targets of Cardioprotection,” Redox Biology 67 (2023): 102894. 10.1016/j.redox.2023.102894 PMC10590874 37839355

[44] B. Drenger , I. A. Ostrovsky , M. Barak , Y. Nechemia‐Arbely , E. Ziv , and J. H. Axelrod , “Diabetes Blockade of Sevoflurane Postconditioning Is Not Restored by Insulin in the Rat Heart: Phosphorylated Signal Transducer and Activator of Transcription 3‐ and Phosphatidylinositol 3‐Kinase‐Mediated Inhibition,” Anesthesiology 114 (2011): 1364–1372. 10.1097/ALN.0b013e31820efafd 21368653

[45] R. Habimana , J. Seong , Y. Jo , et al., “Endotoxin Pretreatment Mitigates Myocardial Ischemia‐Reperfusion Injury Through Preservation of Mitochondrial Respiration: A Combined Assessment of In Vivo, Ex Vivo, and In Vitro Data,” International Journal of Molecular Sciences 26 (2025): 11162. 10.3390/ijms262211162 PMC12653926 41303646

[46] L. Kindernay , V. Farkasova , J. Neckar , J. Hrdlicka , K. Ytrehus , and T. Ravingerova , “Impact of Maturation on Myocardial Response to Ischemia and the Effectiveness of Remote Preconditioning in Male Rats,” International Journal of Molecular Sciences 22 (2021): 11009. 10.3390/ijms222011009 PMC8540346 34681669

[47] N. Wu , L. Wang , S. Yu , et al., “The Novel Sphingosine‐1‐Phosphate Receptor Modulator KRP‐203 Prevents Myocardial Ischemia‐Reperfusion Injury by Preserving Mitochondrial Function Through Activation of the RISK and SAFE Signaling Pathways,” Cell Biology and Toxicology 41 (2025): 159. 10.1007/s10565-025-10114-7 PMC12660407 41307722

[48] K. Boengler , A. Buechert , Y. Heinen , et al., “Cardioprotection by Ischemic Postconditioning Is Lost in Aged and STAT3‐Deficient Mice,” Circulation Research 102 (2008): 131–135. 10.1161/CIRCRESAHA.107.164699 17967780

[49] G. Heusch , J. Musiolik , N. Gedik , and A. Skyschally , “Mitochondrial STAT3 Activation and Cardioprotection by Ischemic Postconditioning in Pigs With Regional Myocardial Ischemia/Reperfusion,” Circulation Research 109 (2011): 1302–1308. 10.1161/CIRCRESAHA.111.255604 21980124

[50] K. Boengler , E. Ungefug , G. Heusch , and R. Schulz , “The STAT3 Inhibitor Stattic Impairs Cardiomyocyte Mitochondrial Function Through Increased Reactive Oxygen Species Formation,” Current Pharmaceutical Design 19 (2013): 6890–6895. 10.2174/138161281939131127115940 23590160

[51] P. Kleinbongard , “Perspective: Mitochondrial STAT3 in Cardioprotection,” Basic Research in Cardiology 118 (2023): 32. 10.1007/s00395-023-01003-3 PMC10449977 37620559

[52] H. R. Lieder , P. Kleinbongard , A. Skyschally , H. Hagelschuer , W. M. Chilian , and G. Heusch , “Vago‐Splenic Axis in Signal Transduction of Remote Ischemic Preconditioning in Pigs and Rats,” Circulation Research 123 (2018): 1152–1163. 10.1161/CIRCRESAHA.118.313859 PMC7304918 30359199

[53] M. Kurdi , C. Zgheib , and G. W. Booz , “Recent Developments on the Crosstalk Between STAT3 and Inflammation in Heart Function and Disease,” Frontiers in Immunology 9 (2018): 3029. 10.3389/fimmu.2018.03029 PMC6305745 30619368

[54] A. Skyschally , S. Gent , G. Amanakis , C. Schulte , P. Kleinbongard , and G. Heusch , “Across‐Species Transfer of Protection by Remote Ischemic Preconditioning With Species‐Specific Myocardial Signal Transduction by Reperfusion Injury Salvage Kinase and Survival Activating Factor Enhancement Pathways,” Circulation Research 117 (2015): 279–288. 10.1161/CIRCRESAHA.117.306878 26058828

[55] H. A. Hildebrandt , V. Kreienkamp , S. Gent , P. Kahlert , G. Heusch , and P. Kleinbongard , “Kinetics and Signal Activation Properties of Circulating Factor(s) From Healthy Volunteers Undergoing Remote Ischemic Pre‐Conditioning,” JACC: Basic to Translational Science 1 (2016): 3–13. 10.1016/j.jacbts.2016.01.007 PMC5012372 27642642

[56] J. Zhou , T. Du , B. Li , Y. Rong , A. Verkhratsky , and L. Peng , “Crosstalk Between MAPK/ERK and PI3K/AKT Signal Pathways During Brain Ischemia/Reperfusion,” ASN Neuro 7 (2015): 7. 10.1177/1759091415602463 PMC4601130 26442853

[57] A. Skyschally , P. Kleinbongard , H. Lieder , et al., “Humoral Transfer and Intramyocardial Signal Transduction of Protection by Remote Ischemic Perconditioning in Pigs, Rats, and Mice,” American Journal of Physiology. Heart and Circulatory Physiology 315 (2018): H159–H172. 10.1152/ajpheart.00152.2018 29569956

[58] S. Gent , A. Skyschally , P. Kleinbongard , and G. Heusch , “Lschemic Preconditioning in Pigs: A Causal Role for Signal Transducer and Activator of Transcription 3,” American Journal of Physiology. Heart and Circulatory Physiology 312 (2017): H478–H484. 10.1152/ajpheart.00749.2016 28039203

[59] L. Barile , E. Cervio , V. Lionetti , et al., “Cardioprotection by Cardiac Progenitor Cell‐Secreted Exosomes: Role of Pregnancy‐Associated Plasma Protein‐A,” Cardiovascular Research 114 (2018): 992–1005. 10.1093/cvr/cvy055 29518183

[60] M. Bartekova , M. Jelemensky , and N. S. Dhalla , “Emerging Role of Non‐Coding RNAs and Extracellular Vesicles in Cardioprotection by Remote Ischemic Conditioning of the Heart,” Reviews in Cardiovascular Medicine 20 (2019): 59–71. 10.31083/j.rcm.2019.02.54 31344998

[61] M. S. Self , K. R. Drasbek , and K. Vissing , “Remote Ischemic Preconditioning and Exercise‐Induced Cardioprotection via Extracellular Vesicle‐Delivered microRNAs,” American Journal of Physiology. Heart and Circulatory Physiology 331 (2026): H39–H57. 10.1152/ajpheart.00954.2025 42166636

[62] D. Gustafson , S. Veitch , and J. E. Fish , “Extracellular Vesicles as Protagonists of Diabetic Cardiovascular Pathology,” Frontiers in Cardiovascular Medicine 4 (2017): 71. 10.3389/fcvm.2017.00071 PMC5701646 29209616

[63] S. Liu , Z. Pan , X. Chen , et al., “The Emerging Role of Extracellular Vesicles in Diabetes and Complications: Mechanistic Insights and Translational Prospects,” Frontiers in Endocrinology 16 (2025): 1674028. 10.3389/fendo.2025.1674028 PMC12537412 41127521

[64] J. Wider , V. V. R. Undyala , P. Whittaker , J. Woods , X. Chen , and K. Przyklenk , “Remote Ischemic Preconditioning Fails to Reduce Infarct Size in the Zucker Fatty Rat Model of Type‐2 Diabetes: Role of Defective Humoral Communication,” Basic Research in Cardiology 113 (2018): 16. 10.1007/s00395-018-0674-1 PMC6776086 29524006

[65] J. Laura Frances , C. Pagiatakis , V. Di Mauro , and M. Climent , “Therapeutic Potential of EVs: Targeting Cardiovascular Diseases,” Biomedicine 11 (2023): 1907. 10.3390/biomedicines11071907 PMC10377624 37509546

[66] A. Dushpanova , E. Redolfi‐Riva , V. Casieri , et al., “Reconnecting the Vagus Nerve to the Heart Through Nerve Conduit Preserves Cardiac Function in a Minipig Model of Right Cardiac Vagotomy,” Science Translational Medicine 17 (2025): eaea4306. 10.1126/scitranslmed.aea4306 41442503

[67] R. Chakravarti , P. R. Bankar , J. Chigilipalli , et al., “An Explicative Review on Diabetic Cardiac Autonomic Neuropathy: From Pathophysiology to the Era of Gene Therapy,” Vascular Pharmacology 164 (2026): 107685. 10.1016/j.vph.2026.107685 42562037

[68] S. Comita , S. Femmino , C. Thairi , et al., “Regulation of STAT3 and Its Role in Cardioprotection by Conditioning: Focus on Non‐Genomic Roles Targeting Mitochondrial Function,” Basic Research in Cardiology 116 (2021): 56. 10.1007/s00395-021-00898-0 PMC8510947 34642818

[69] C. Wang , S. Shen , J. Kang , et al., “METTL3 Is Essential for Exercise Benefits in Diabetic Cardiomyopathy,” Circulation 152 (2025): 327–345. 10.1161/CIRCULATIONAHA.124.070279 PMC12324960 40357551

[70] K. Owais , T. Huang , F. Mahmood , et al., “Cardiopulmonary Bypass Decreases Activation of the Signal Transducer and Activator of Transcription 3 (STAT3) Pathway in Diabetic Human Myocardium,” Annals of Thoracic Surgery 100 (2015): 1636–1645. 10.1016/j.athoracsur.2015.05.013 26228595

[71] A. Haghikia , M. Ricke‐Hoch , B. Stapel , I. Gorst , and D. Hilfiker‐Kleiner , “STAT3, a Key Regulator of Cell‐To‐Cell Communication in the Heart,” Cardiovascular Research 102 (2014): 281–289. 10.1093/cvr/cvu034 24518140

[72] K. Shirakawa , J. Endo , M. Kataoka , et al., “IL (Interleukin)‐10‐STAT3‐Galectin‐3 Axis Is Essential for Osteopontin‐Producing Reparative Macrophage Polarization After Myocardial Infarction,” Circulation 138 (2018): 2021–2035. 10.1161/CIRCULATIONAHA.118.035047 29967195

[73] A. Subramani , K. Essandoh , M. Y. Young , et al., “Cardiomyocyte Janus Kinase 1 (JAK1) Signaling Is Required for Cardiac Homeostasis and Cytokine‐Dependent Activation of STAT3,” Journal of Molecular and Cellular Cardiology 207 (2025): 13–23. 10.1016/j.yjmcc.2025.07.017 PMC12370008 40744288

[74] F. A. Zouein , M. Kurdi , and G. W. Booz , “LIF and the Heart: Just Another Brick in the Wall?,” European Cytokine Network 24 (2013): 11–19. 10.1684/ecn.2013.0335 PMC3674483 23661360

[75] Y. Zheng , Y. Wang , B. Qi , et al., “IL6/Adiponectin/HMGB1 Feedback Loop Mediates Adipocyte and Macrophage Crosstalk and M2 Polarization After Myocardial Infarction,” Frontiers in Immunology 15 (2024): 1368516. 10.3389/fimmu.2024.1368516 PMC11004445 38601146

[76] M. Pipicz , V. Demjan , M. Sarkozy , and T. Csont , “Effects of Cardiovascular Risk Factors on Cardiac STAT3,” International Journal of Molecular Sciences 19 (2018): 3572. 10.3390/ijms19113572 PMC6274853 30424579

[77] L. Wu , W. Q. Huang , C. C. Yu , and Y. F. Li , “Moderate Hydrogen Peroxide Postconditioning Ameliorates Ischemia/Reperfusion Injury in Cardiomyocytes via STAT3‐Induced Calcium, ROS, and ATP Homeostasis,” Pharmacology 106 (2021): 275–285. 10.1159/000511961 33302272

[78] Z. Chen and Z. C. Han , “STAT3: A Critical Transcription Activator in Angiogenesis,” Medicinal Research Reviews 28 (2008): 185–200. 10.1002/med.20101 17457812

[79] A. Bea , J. G. Valero , A. Irazoki , et al., “Cardiac Fibroblasts Display Endurance to Ischemia, High ROS Control and Elevated Respiration Regulated by the JAK2/STAT Pathway,” FEBS Journal 289 (2022): 2540–2561. 10.1111/febs.16283 34796659

[80] H. H. Guo , X. Y. Jing , H. Chen , H. X. Xu , and B. M. Zhu , “STAT3 but Not STAT5 Contributes to the Protective Effect of Electroacupuncture Against Myocardial Ischemia/Reperfusion Injury in Mice,” Frontiers in Medicine 8 (2021): 649654. 10.3389/fmed.2021.649654 PMC8299366 34307396

[81] P. Kleinbongard , A. Skyschally , S. Gent , M. Pesch , and G. Heusch , “STAT3 as a Common Signal of Ischemic Conditioning: A Lesson on ‘Rigor and Reproducibility’ in Preclinical Studies on Cardioprotection,” Basic Research in Cardiology 113 (2018): 3, 10.1007/s00395-017-0660-z. 29159507

[82] G. Heusch , J. Musiolik , E. Kottenberg , J. Peters , H. Jakob , and M. Thielmann , “STAT5 Activation and Cardioprotection by Remote Ischemic Preconditioning in Humans: Short Communication,” Circulation Research 110 (2012): 111–115. 10.1161/CIRCRESAHA.111.259556 22116817

[83] A. Padmanabhan , M. Alexanian , R. Linares‐Saldana , et al., “BRD4 (Bromodomain‐Containing Protein 4) Interacts With GATA4 (GATA Binding Protein 4) to Govern Mitochondrial Homeostasis in Adult Cardiomyocytes,” Circulation 142 (2020): 2338–2355. 10.1161/CIRCULATIONAHA.120.047753 PMC7736290 33094644

[84] L. Zhuang , K. Jia , C. Chen , et al., “DYRK1B‐STAT3 Drives Cardiac Hypertrophy and Heart Failure by Impairing Mitochondrial Bioenergetics,” Circulation 145 (2022): 829–846. 10.1161/CIRCULATIONAHA.121.055727 35235343

[85] M. Mericskay , C. J. Zuurbier , L. C. Heather , et al., “Cardiac Intermediary Metabolism in Heart Failure: Substrate Use, Signalling Roles and Therapeutic Targets,” Nature Reviews. Cardiology 22 (2025): 704–727.10.1038/s41569-025-01166-740544173

[86] S. S. Jankauskas , U. Kansakar , F. Varzideh , et al., “Heart Failure in Diabetes,” Metabolism 125 (2021): 154910. 10.1016/j.metabol.2021.154910 PMC8941799 34627874

[87] K. Boengler , D. Hilfiker‐Kleiner , G. Heusch , and R. Schulz , “Inhibition of Permeability Transition Pore Opening by Mitochondrial STAT3 and Its Role in Myocardial Ischemia/Reperfusion,” Basic Research in Cardiology 105 (2010): 771–785. 10.1007/s00395-010-0124-1 PMC2978889 20960209

[88] C. Du , Y. Zhu , J. Duan , et al., “A‐485 Alleviates Fibrosis and Apoptosis in Kidney by Disrupting Tandem Activation of Acetylation and Phosphorylation on STAT3,” Biomedicine & Pharmacotherapy 188 (2025): 118217. 10.1016/j.biopha.2025.118217 40435839

[89] Y. Gao , Z. Zhao , X. Chen , et al., “RNF220 Mediates K63‐Linked Polyubiquitination of STAT3 and Aggravates Pathological Cardiac Hypertrophy,” Cell Death and Differentiation 33 (2026): 945–958. 10.1038/s41418-025-01614-4 PMC13156277 41219491

[90] G. Kundu , M. Ghasemi , S. Yim , et al., “STAT3 Protein‐Protein Interaction Analysis Finds P300 as a Regulator of STAT3 and Histone 3 Lysine 27 Acetylation in Pericytes,” Biomedicine 12 (2024): 12. 10.3390/biomedicines12092102 PMC11428717 39335615

[91] D. Hilfiker‐Kleiner , K. Kaminski , E. Podewski , et al., “A Cathepsin D‐Cleaved 16 kDa Form of Prolactin Mediates Postpartum Cardiomyopathy,” Cell 128 (2007): 589–600. 10.1016/j.cell.2006.12.036 17289576

[92] A. Saleh , S. K. Chowdhury , D. R. Smith , et al., “Diabetes Impairs an Interleukin‐1beta‐Dependent Pathway That Enhances Neurite Outgrowth Through JAK/STAT3 Modulation of Mitochondrial Bioenergetics in Adult Sensory Neurons,” Molecular Brain 6 (2013): 45. 10.1186/1756-6606-6-45 PMC4016027 24152426

[93] M. K. Tawfik , D. I. Badran , M. M. Keshawy , S. Makary , and M. Abdo , “Alternate‐Day Fat Diet and Exenatide Modulate the Brain Leptin JAK2/STAT3/SOCS3 Pathway in a Fat Diet‐Induced Obesity and Insulin Resistance Mouse Model,” Archives of Medical Science 19 (2023): 1508–1519. 10.5114/aoms/158534 PMC10507768 37732053

[94] E. N. Gurzov , W. J. Stanley , E. G. Pappas , H. E. Thomas , and D. J. Gough , “The JAK/STAT Pathway in Obesity and Diabetes,” FEBS Journal 283 (2016): 3002–3015. 10.1111/febs.13709 26972840

[95] I. J. Marie , T. Lahiri , O. Onder , K. S. J. Elenitoba‐Johnson , and D. E. Levy , “Structural Determinants of Mitochondrial STAT3 Targeting and Function,” Mitochondrial Communications 2 (2024): 1–13. 10.1016/j.mitoco.2024.01.001 PMC10947224 38500969

[96] R. C. Sloan , F. Moukdar , C. R. Frasier , et al., “Mitochondrial Permeability Transition in the Diabetic Heart: Contributions of Thiol Redox State and Mitochondrial Calcium to Augmented Reperfusion Injury,” Journal of Molecular and Cellular Cardiology 52 (2012): 1009–1018. 10.1016/j.yjmcc.2012.02.009 22406429

[97] P. Kleinbongard , G. Amanakis , A. Skyschally , and G. Heusch , “Reflection of Cardioprotection by Remote Ischemic Perconditioning in Attenuated ST‐Segment Elevation During Ongoing Coronary Occlusion in Pigs: Evidence for Cardioprotection From Ischemic Injury,” Circulation Research 122 (2018): 1102–1108, 10.1161/CIRCRESAHA.118.312784. 29467197

[98] L. Alibrandi and V. Lionetti , “Interspecies Differences in Mitochondria: Implications for Cardiac and Vascular Translational Research,” Vascular Pharmacology 159 (2025): 107476. 10.1016/j.vph.2025.107476 40037508

